# Supplementary material for: Plasmonic monolithic lithium niobate directional coupler switches
Source: Nat Commun. 2020 Feb 6;11:748. doi: 10.1038/s41467-020-14539-y (PMC7005156; doi:10.1038/s41467-020-14539-y)
Supplement: Supplementary file 3 — Supplementary Information [file 41467_2020_14539_MOESM3_ESM.pdf]

Supplementary Information

Plasmonic monolithic lithium niobate directional coupler switches

Thomaschewski et al.

Supplementary Note 1: Coupled mode formalism

The evolution of a wave (mode), propagating along y-axis with the propagation constant of β, can be described as following (here for the electromagnetic wave):

$$\{\mathbf{E}(x,y,z),\mathbf{H}(x,y,z)\} = \{\mathbf{E}(x,z),\mathbf{H}(x,z)\}C\exp(j\beta y) \tag{1.1}$$

When there are two non-interacting waveguides, each carrying a single mode with a complex-valued amplitude of C_1 and C_2, respectively, the total field can be written in a similar form:

$$\{\mathbf{E}(x,y,z),\mathbf{H}(x,y,z)\} = \{\mathbf{E_1}(x,z),\mathbf{H_1}(x,z)\}C_1\exp(j\beta_1 y) + \{\mathbf{E_2}(x,z),\mathbf{H_2}(x,z)\}C_2\exp(j\beta_2 y) \tag{1.2}$$

This should be understood as following: the fields in the area of the first waveguides are described only with the first term (i.e., the field from the second mode does not reach this area), and the second term describes the fields in the area of the second waveguide. However, when waveguides are brought together, there is non-zero overlap between their modes, therefore they couple, i.e., the power transfers from one mode into the other. If the coupling is relatively weak, one can still use the above equation 1.2, but with the assumption that the mode amplitudes C_1 and C_2 are no longer constants, but instead have a slow evolution along the propagation axis y, which can be described with the following differential equations:[1,2]

$$\begin{cases} C_1{}' = j\kappa C_2\exp(-j\Delta\beta y), \\ C_2{}' = j\kappa C_1\exp(j\Delta\beta y), \end{cases} \tag{1.3}$$

where the primes denote differentiation with respect to the propagation direction (y-axis), $\Delta\beta = \beta_1 - \beta_2$ is the difference in the propagation constants of the modes, and κ is the coupling coefficient. Basically, the above equation tells that the modification of one mode amplitude is proportional to the relative amplitude of the other mode. Using a substitution of

$$\begin{cases} R(y) \equiv C_1(y)\exp\left(\frac{1}{2}j\Delta\beta y\right), \\ S(y) \equiv C_2(y)\exp\left(-\frac{1}{2}j\Delta\beta y\right), \end{cases} \tag{1.4}$$

the above equation is modified to the following:

$$\begin{cases} R' = \frac{1}{2}j\Delta\beta R + j\kappa S, \\ S' = j\kappa R - \frac{1}{2}j\Delta\beta S, \end{cases} \tag{1.5}$$

At the same time equation 1.2 will get the following form:

$$\{\mathbf{E}(x,y,z),\mathbf{H}(x,y,z)\} = [\{\mathbf{E_1}(x,z),\mathbf{H_1}(x,z)\}R(y) + \{\mathbf{E_2}(x,z),\mathbf{H_2}(x,z)\}S(y)]\exp\left(j\beta_{\text{avg}} y\right), \tag{1.6}$$

with the average propagation constant $\beta_{\mathrm{avg}} = (\beta_1 + \beta_2)/2$ of the two modes. In order to solve Eq. 1.5, we search for a solution in the form $R/S = \alpha \equiv \mathrm{const}$. Then Eq. 1.5 transforms into

$$\begin{cases} \alpha S' = \frac{1}{2} j\Delta\beta\alpha S + j\kappa S, \\ S' = j\kappa\alpha S - \frac{1}{2} j\Delta\beta S; \end{cases}$$

$$\alpha\left(j\kappa\alpha - \tfrac{1}{2} j\Delta\beta\right) = \tfrac{1}{2} j\Delta\beta\alpha + j\kappa;$$

$$\alpha^2 - \frac{\Delta\beta}{\kappa}\alpha - 1 = 0;$$

$$\begin{cases} \alpha_{a,b} = \frac{\Delta\beta}{2\kappa} \pm \sqrt{1 + \left(\frac{\Delta\beta}{2\kappa}\right)^2} \equiv \Delta\beta_{\mathrm{n}} \pm \gamma, \\ \alpha_a \alpha_b = -1, \end{cases} \quad (1.7)$$

where $\Delta\beta_{\mathrm{n}} = \frac{\Delta\beta}{2\kappa}$, $\gamma = \sqrt{1 + \Delta\beta_{\mathrm{n}}{}^2}$. Using such α, one can get a general solution of Eq. 1.5 for R and S:

$$\begin{cases} S(y) = S_a \exp\left(j\left[\kappa\alpha_a - \frac{1}{2}\Delta\beta\right] y\right) + S_b \exp\left(j\left[\kappa\alpha_b - \frac{1}{2}\Delta\beta\right] y\right), \\ R(y) = \alpha_a S_a \exp\left(j\left[\kappa\alpha_a - \frac{1}{2}\Delta\beta\right] y\right) + \alpha_b S_b \exp\left(j\left[\kappa\alpha_b - \frac{1}{2}\Delta\beta\right] y\right), \end{cases} \quad (1.8)$$

where S_a and S_b are constants, determined from initial (boundary) conditions. One can see that each of the above two solutions represents a true mode of the system of two coupled waveguides, since then Eq. 1.6 will have the same form as for the single mode (Eq. 1.1):

$$\{\mathbf{E}(x,y,z), \mathbf{H}(x,y,z)\} = [\{\mathbf{E_1}(x,z), \mathbf{H_1}(x,z)\}\alpha + \{\mathbf{E_2}(x,z), \mathbf{H_2}(x,z)\}] S_\alpha e^{j\left(\kappa\alpha - \frac{1}{2}\Delta\beta + \beta_{\mathrm{avg}}\right)y}, \quad (1.9)$$

with the propagation constant of the mode $\beta = \beta_{\mathrm{avg}} + \kappa\alpha - \frac{1}{2}\Delta\beta$. When both waveguides are identical ($\Delta\beta = 0$), the ratio $R/S = \alpha$ is either 1 or -1 (see Eq. 1.7), therefore these supermodes are called even (symmetric) and odd (asymmetric). Their propagation constants are thus:

$$\begin{cases} \alpha_{\mathrm{even}} = \Delta\beta_{\mathrm{n}} + \gamma, \quad \alpha_{\mathrm{odd}} = \Delta\beta_{\mathrm{n}} - \gamma, \\ \beta_{\mathrm{even}} = \beta_{\mathrm{avg}} + \kappa\gamma, \quad \beta_{\mathrm{odd}} = \beta_{\mathrm{avg}} - \kappa\gamma. \end{cases} \quad (1.10)$$

Thus the CMT predicts the following conclusions: 1) the difference between propagation constants of even and odd mode (so-called splitting) is increasing with the increase of the mismatch between waveguides propagation constants ($\Delta\beta$) independently on its sign; 2) the mode with larger propagation constant tends to concentrate at the waveguide with larger propagation constant (for example, if $\kappa > 0$ and $\Delta\beta > 0$, then $\beta_{\mathrm{even}} > \beta_{\mathrm{odd}}$, but also $\alpha_{\mathrm{even}} > 1$, i.e., there is more power of the even mode in the first guide, which has larger propagation constant), and the other mode has the opposite tendency. The last is especially obvious for

the case of electro-optic modulation, where only the refractive index of waveguide environment is modulated: if even or odd mode tends to concentrate more in the place with increased refractive index, then its propagation constant should also be increased, compared to the unmodulated case. Finally, when both waveguides are identical ($\Delta\beta = 0$), the coupling coefficient can be expressed as

$$\kappa = (\beta_{\text{even}} - \beta_{\text{odd}})/2 \tag{1.11}$$

The amplitude distribution in each guide (Eq. 1.8) is convenient to express in terms of their initial values $R(y = 0) \equiv R_0$ and $S(y = 0) \equiv S_0$:

$$\begin{cases} S_a = \dfrac{R_0 - \alpha_b S_0}{\alpha_a - \alpha_b}, \\ S_b = \dfrac{\alpha_a S_0 - R_0}{\alpha_a - \alpha_b}; \end{cases}$$

$$\begin{cases} R(y) = \alpha_a S_a \exp(j\kappa\gamma y) + \alpha_b S_b \exp(-j\kappa\gamma y), \\ S(y) = S_a \exp(j\kappa\gamma y) + S_b \exp(-j\kappa\gamma y); \end{cases}$$

$$\begin{cases} R(y) = R_0 \cos(\kappa\gamma y) + j\left[\dfrac{\Delta\beta_{\text{n}}}{\gamma} R_0 + \dfrac{1}{\gamma} S_0\right] \sin(\kappa\gamma y), \\ S(y) = S_0 \cos(\kappa\gamma y) + j\left[\dfrac{1}{\gamma} R_0 - \dfrac{\Delta\beta_{\text{n}}}{\gamma} S_0\right] \sin(\kappa\gamma y). \end{cases}$$

The power distribution is then:

$$\begin{cases} |R(y)|^2 = |R_0 \cos(\kappa\gamma y)|^2 + \left|\dfrac{\Delta\beta_{\text{n}} R_0 + S_0}{\gamma} \sin(\kappa\gamma y)\right|^2 + 2\text{Im}\left\{R_0 \cos(\kappa\gamma y)\left[\dfrac{\Delta\beta_{\text{n}} R_0 + S_0}{\gamma} \sin(\kappa\gamma y)\right]^*\right\}, \\ |S(y)|^2 = |S_0 \cos(\kappa\gamma y)|^2 + \left|\dfrac{R_0 - \Delta\beta_{\text{n}} S_0}{\gamma} \sin(\kappa\gamma y)\right|^2 + 2\text{Im}\left\{S_0 \cos(\kappa\gamma y)\left[\dfrac{R_0 - \Delta\beta_{\text{n}} S_0}{\gamma} \sin(\kappa\gamma y)\right]^*\right\}. \end{cases}$$

where the asterisk indicates a complex conjugate, and Im{} means an operator for taking the imaginary part. When all values are real, then the last term in each row of the above equation is zero, thus simplifying the equation for the power distribution:

$$\begin{cases} |R|^2 = R_0^2 \cos^2(\kappa\gamma y) + \left[\frac{\Delta\beta_{\text{n}} R_0 + S_0}{\gamma}\right]^2 \sin^2(\kappa\gamma y), \\ |S|^2 = S_0^2 \cos^2(\kappa\gamma y) + \left[\frac{R_0 - \Delta\beta_{\text{n}} S_0}{\gamma}\right]^2 \sin^2(\kappa\gamma y). \end{cases} \tag{1.12}$$

Conventional directional coupler. If light is launched only in one channel of identical waveguides ($R_0 = 1$, $S_0 = 0$), and if no modulation is applied ($\Delta\beta = 0$), then the first complete switch ($R = 0$, $S = 1$) will appear at the length L, where $\kappa\gamma L = \pi/2$, i.e, at $L = \pi/(2\kappa\gamma) = \pi/(2\kappa)$ (See Eq. 1.12). This interaction length is called a coupling length:

$$L_{\mathrm{C}} \equiv \frac{\pi}{2\kappa} = \frac{\pi}{\beta_{\mathrm{even}}(V=0)-\beta_{\mathrm{odd}}(V=0)}, \qquad (1.13)$$

where the propagation coefficients of even and odd modes are calculated without applied voltage.

From *even/odd mode formalism* the operation of a conventional directional coupler can be seen as following: both even and odd modes are excited with the same amplitude and phase, resulting in the constructive interference in the channel R (R_0 = 1) and destructive interference in the channel S (S_0 = 0). After propagating a length of L_{C} these modes get a phase mismatch of π due to different propagation constants, which will result in R = 0, S = 1 (i.e., destructive interference in channel R and constructive interference in channel S).

If such a device (with $L = L_{\mathrm{C}}$) is used for modulation ($\Delta\beta \neq 0$), then the complete switch back (R = 1, S = 0) can be achieved at $\kappa\gamma L_{\mathrm{C}} = \pi$ (according to Eq. 1.12), i.e., at $\gamma = 2$, which can be achieved at the modulation $\Delta\beta_{\mathrm{n}} = \sqrt{3}$. The phase mismatch, achieved in such a device upon full switch, is thus $\Delta\beta L = 2\kappa\Delta\beta_{\mathrm{n}} L_{\mathrm{C}} = \sqrt{3}\pi$. A commonly reported number for waveguide-modulator design is the voltage V_{π}, required to produce the phase mismatch of π. Since $\Delta\beta$ is linearly proportional to the applied voltage (for small modifications of the refractive index), then the full-switch voltage of the conventional directional coupler is thus $V_{\mathrm{full\,switch}} = \sqrt{3}V_{\pi}$.

From *even/odd mode formalism* the operation of a modulated directional coupler can be seen as following: the launching condition (R_0 = 1, S_0 = 0) result in the excitation of both these modes, with amplitudes resulting in a completely destructive interference in channel S (similarly to unmodulated case, but now amplitudes of even and odd modes are not the same due to the electrically induced asymmetry). If these modes acquire a phase mismatch of 2π after propagating the device length ($L = L_{\mathrm{C}}$), then at the output they will result in the same interference condition as at the input, i.e., $|R|$ = 1, $|S|$ = 0, meaning the complete switch back. This will happen when the difference in the propagation constants of even and odd modes upon modulation is twice as large compared to the unmodulated case. From Eq. 1.10 it means $\gamma = 2$, agreeing exactly with the above derivations from CMT.

Our directional coupler. In our design both channels are equally launched ($R_0 = S_0 = 1/\sqrt{2}$). Then the optical power $|R|^2$ and $|S|^2$in two waveguides are

$$\begin{cases} |R|^2 = \frac{1}{2}\left[1 + \frac{2\Delta\beta_{\mathrm{n}}}{\gamma^2}\sin^2\left(\frac{\pi}{2}L_{\mathrm{n}}\gamma\right)\right] \equiv P_1, \\ |S|^2 = \frac{1}{2}\left[1 - \frac{2\Delta\beta_{\mathrm{n}}}{\gamma^2}\sin^2\left(\frac{\pi}{2}L_{\mathrm{n}}\gamma\right)\right] \equiv P_2, \end{cases} \qquad (1.14)$$

where $L_{\mathrm{n}} \equiv \frac{L}{L_{\mathrm{C}}} = \frac{2\kappa L}{\pi}$ is the normalized interaction length. Using a direct expression for γ, one can obtain equation (1) in the main text:

$$P_{1,2} = \frac{1}{2}\left[1 \pm \frac{2}{\Delta\beta_{\mathrm{n}} + \frac{1}{\Delta\beta_{\mathrm{n}}}}\sin^2\left(\frac{\pi}{2}L_{\mathrm{n}}\sqrt{1 + \Delta\beta_{\mathrm{n}}^{\,2}}\right)\right] \qquad (1.15)$$

With no modulation voltage applied, the coupler is inherently operating at its 3 dB point due to the symmetry of the system. By applying a voltage, light can be coupled from one channel into the other by introducing an electro-optically induced mismatch $\Delta\beta$ between the propagation constants of the coupled waveguides. The full off-state in one channel (when P_1 or P_2 equal to zero) can be reached when $|\Delta\beta_{\mathrm{n}}| = 1$ (i.e., the required voltage should change propagation constant of each channel to the same amount as the difference between those of the even and odd mode at no modulation), and when the sine function is at maximum amplitude (i.e., $\frac{\pi}{2}L_{\mathrm{n}}\sqrt{1+\Delta\beta_{\mathrm{n}}{}^{2}} = \frac{\pi}{2} + \pi m$, $\sqrt{2}L_{\mathrm{n}} = 1 + 2m$, where m is an integer number, $m \in \mathbb{Z}$). Thus, the smallest interaction length required for the complete switch is $L = L_{\mathrm{C}}/\sqrt{2}$. It might seem that our device is superior to a conventional directional coupler in both shorter device length (by a factor of $\sqrt{2}$) and smaller voltage (by a factor of $\sqrt{3}$) required for the complete switch. However, the full-switch voltage should be calculated for the transition from the complete off-state to the complete on-state (e.g., from $P_1 = 0, P_2 = 1$ to $P_1 = 1, P_2 = 0$), and not from the balanced 3 dB point as in our device at no modulation. Thus, the condition for the complete switch is $|\Delta\beta_{\mathrm{n}}| = 2$, corresponding to the phase mismatch of $\Delta\beta L = 2\kappa\Delta\beta_{\mathrm{n}}L_{\mathrm{C}}/\sqrt{2} = \sqrt{2}\pi$. The full-switch voltage is then $V_{\mathrm{full\,switch}} = \sqrt{2}V_{\pi}$, which is still smaller than the one for a conventional directional coupler.

From *even/odd mode formalism* the operation of our directional coupler can be seen as following: when no modulation is applied, only the even mode is excited, therefore the power distribution in both guides is the same at any point along the propagation (*R* = *S*). However, when the voltage is applied, the initial condition does not satisfy the excitation of only the even mode (since the refractive index distribution becomes uneven, so even and odd modes are not completely even and odd in terms of field distribution – see Eq. 1.10). A complete off-state in one of the channels (R or S) can be achieved when contributions from even and odd modes to this channel interfere destructively, i.e., they equal in amplitude, but their phase mismatch is $\pi + \pi m, m \in \mathbb{Z}$. One can show that the condition for equal amplitudes is achieved when the asymmetry coefficient α of even/odd mode is $1\pm\sqrt{2}$. Comparing with Eq. 1.7 this means $|\Delta\beta_{\mathrm{n}}| = 1$, agreeing to the above derivations from CMT. The condition for the opposite phase between even and odd modes results in the following: $[\beta_{\mathrm{odd}}(V) - \beta_{\mathrm{even}}(V)]L = \pi + 2\pi m, m \in \mathbb{Z}$. Using Eq. 1.10 and Eq. 1.13, it will transform into $\pi L_{\mathrm{n}}\gamma = \pi + 2\pi m$, or $\sqrt{2}L_{\mathrm{n}} = 1 + 2m$ (since $\gamma = \sqrt{2}$ for $|\Delta\beta_{\mathrm{n}}| = 1$). This is exactly the same condition for the normalized interaction length as derived from CMT. Note that at this condition the difference in modified propagation constants of even and odd modes is increased by a factor of $\sqrt{2}$, compared to the unmodulated one: $\beta_{\mathrm{even}}(V) - \beta_{\mathrm{odd}}(V) = \sqrt{2}[\beta_{\mathrm{even}}(V=0) - \beta_{\mathrm{odd}}(V=0)]$.

Supplementary Figure 1 below shows the output power $P_{1,2}$ in each channel versus the normalized interaction length L_{n} for different $\Delta\beta_{\mathrm{n}}$, calculated with Eq. 1.15. The absolute maximum modulation depth (full switch-off in P_2) is achieved when $\Delta\beta_{\mathrm{n}} = 1$ and $L_{\mathrm{n}} = (1+2m)/\sqrt{2}$, as predicted above. However, if such modulation

$\Delta\beta$ cannot be achieved (for example, due to the limitations in voltage supply or electric breakdown of the device), strongest modulation is achieved for a different device length, when $L_\mathrm{n} = 1/\sqrt{1 + \Delta\beta_\mathrm{n}^{\;2}}$, i.e., when the sine function in Eq. 1.15 reaches 1. Finally, one can note that for positive modulation $\Delta\beta_\mathrm{n} > 0$ plotted in Supplementary Figure 1 (meaning $\beta_1 > \beta_2$) the power in the first channel is always larger than the one in the second channel ($P_1 \geq P_2$). This can be intuitively understood by the light's tendency to leak towards optically denser medium, which is waveguide 1 in our case.

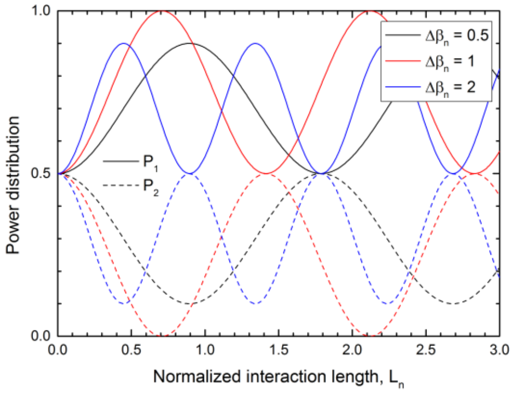

Supplementary Figure 1. Output power P_1 (solid) and P_2 (dashed) versus the normalized interaction length L_n for different $\Delta\beta_\mathrm{n}$ = 0.5 (black), 1 (red), 2 (blue). In our experimental study, the directional coupler satisfies the condition of $L_\mathrm{n} = 1$, thus allowing full optical switching.

Mach-Zehnder interferometer. In a usual Mach-Zehnder interferometer a single waveguide splits into two identical waveguides (arms of the interferometer), which after propagating some lengths are combined into a single waveguide. Both splitting and merging acts as a filter for the even mode. That is, after the splitting the power in each arm is the same ($R_0 = S_0$), and at the merging junction the transmitted power into the single waveguide is proportional to $|R + S|$. Therefore, when no modulation is applied, and both interferometers arms are identical, then the transmission is at maximum (100% in the lossless case). In order to switch-off the transmission, one needs to apply the modulation, such that modes R and S at the merging junction will be out-of-phase, i.e., $\Delta\beta L = \pi$. Thus, the full-switch voltage in Mach-Zehnder interferometer device is $V_\text{full switch} = V_\pi$, which is the smallest among studied here devices. The drawbacks of Mach-Zehnder interferometer-based modulator is larger size and extra losses due to Y-junctions and routing required to separate interferometer arms.

Supplementary Note 2: Numerical electrostatic and mode analysis

Numerical simulations accompanying CMT are performed using a finite element method solver, implemented in commercially available software (Comsol Multiphysics 5.2a). In the first step, electrostatic simulations (Supplementary Figures 2a,c) are performed, in which the relative permittivity of air is set to $\varepsilon_{\mathrm{air}} = 1$, and the boundaries of the two gold nanostripes are set to ground and V_{bias} potential, respectively. For simulations at small modulation frequencies (< 1 MHz) we used the unclamped static relative permittivity tensor of LN taken from Jazbinsek et al.[3] ($\varepsilon_{xx} = \varepsilon_{yy} = 84.48$, $\varepsilon_{zz} = 27.78$). At higher frequencies (> 10 MHz) the crystal strain can no longer follow the changes of the external electric field. Thus, the clamped static relative permittivity tensor of LN taken from Jazbinsek et al.[3] ($\varepsilon_{xx} = \varepsilon_{yy} = 45.52$, $\varepsilon_{zz} = 26.22$) is used for the estimation of the device bandwidth (calculated from its capacitance). According to our simulations (see Supplementary Figure 2), there is a negligible difference in the electrostatic field distribution, calculated for unclamped and clamped values of LN permittivity. However, the clamped permittivity of LN reduces the modulation by ~20%, and results in almost 1.5 times smaller capacitance, as compared to the simulations with unclamped values of LN permittivity.

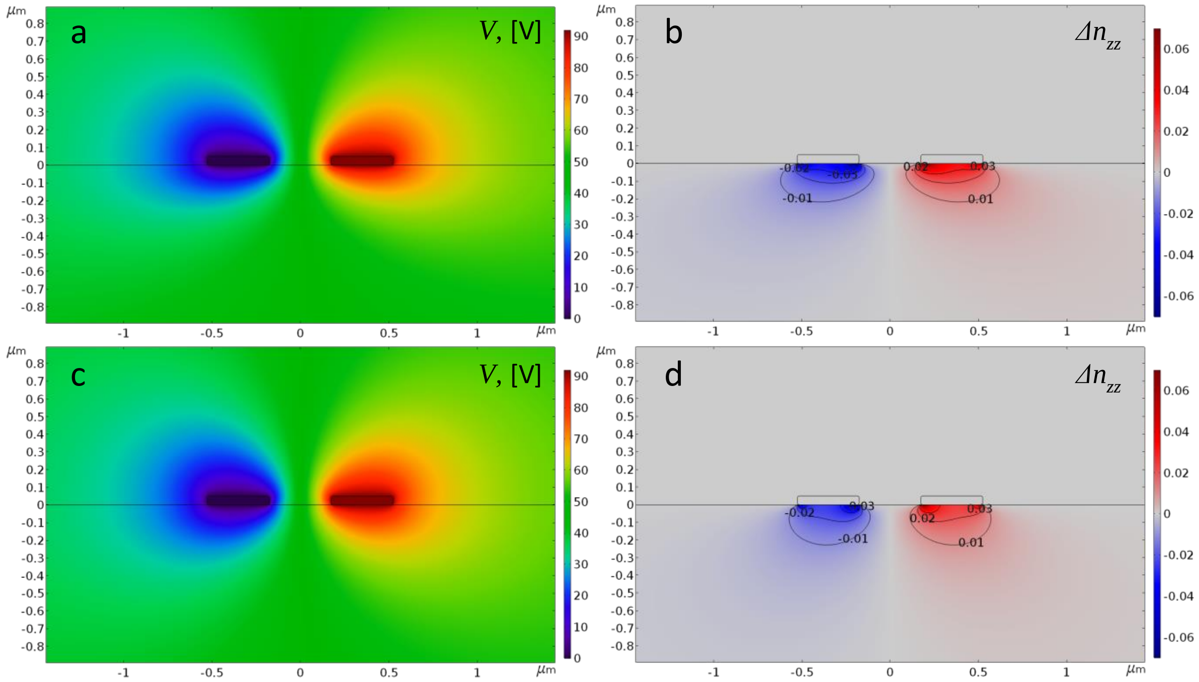

Supplementary Figure 2. Electrostatic simulations upon applying a full-switch voltage (92 V for our device), illustrating the distribution of the (a, c) potential and (b, d) electrically-induced change in the refractive index Δn_{zz} (corresponding to the extraordinary axis of LN substrate), calculated while using (a, b) unclamped and (c, d) clamped permittivity of LN.

The calculated electric-field distribution is utilized to determine the modification of the refractive index in the LN substrate (Supplementary Figures 2b,d) by using the electro-optic Pockels coefficients from Jazbinsek et

al.[3] (restricting to the largest diagonal terms, i.e., $\Delta n_{ii} = -0.5 r_{iiz} n_{ii}^3 E_z$, with $r_{xxz} = r_{yyz} = 10.12$ pm/V and $r_{zzz} = 31.45$ pm/V). For the optical simulation, the modified distribution of the refractive index of LN is fed into the mode solver, while the unmodified refractive index of LN was taken from Zelmon et al.[4] ($n_{xx} = n_{yy} = n_o = 2.211$, $n_{zz} = n_e = 2.138$ at $\lambda_0 = 1550$ nm). The refractive index of gold was taken from Lorentz-Drude (LD) fitting model[5]. A perfectly matched layer is applied on the boundary of the circular simulation domain (with a diameter of 10 μm) in order to absorb optical leakage. To distinguish real modes from cavity modes, the simulation domain size was varied (the effective mode index $N_{\text{eff}} \equiv \frac{\beta}{k_0} = \frac{\lambda\beta}{2\pi}$ and the field distribution of real modes does not depend on the simulation domain size, on the contrary to those of cavity modes). The calculated modes are leaky, because of their effective mode indices are being lower that the ordinary refractive index of LN substrate ($n_o = 2.211$). To estimate the amount of losses due to the leakage, we duplicated simulations with a lossless gold (by setting $\text{Im}[\varepsilon_{\text{Au}}] = 0$). It appeared that the leakage losses are responsible only for a few percent of total losses and is thus neglectable compared to the plasmonic loss.

In order to determine the coupling coefficient κ used in CMT, mode analysis was performed with both metal stripes present and unmodified refractive index of LN substrate (Supplementary Figures 3a,b). The calculated even and odd mode, and their effective mode indices ($n_{\text{even}} = 2.168$ and $n_{\text{odd}} = 2.133$) were used to determine the coupling coefficient κ (using Eq. 1.11) and the corresponding coupling length $L_C = 21.9$ μm (using Eq. 1.13).

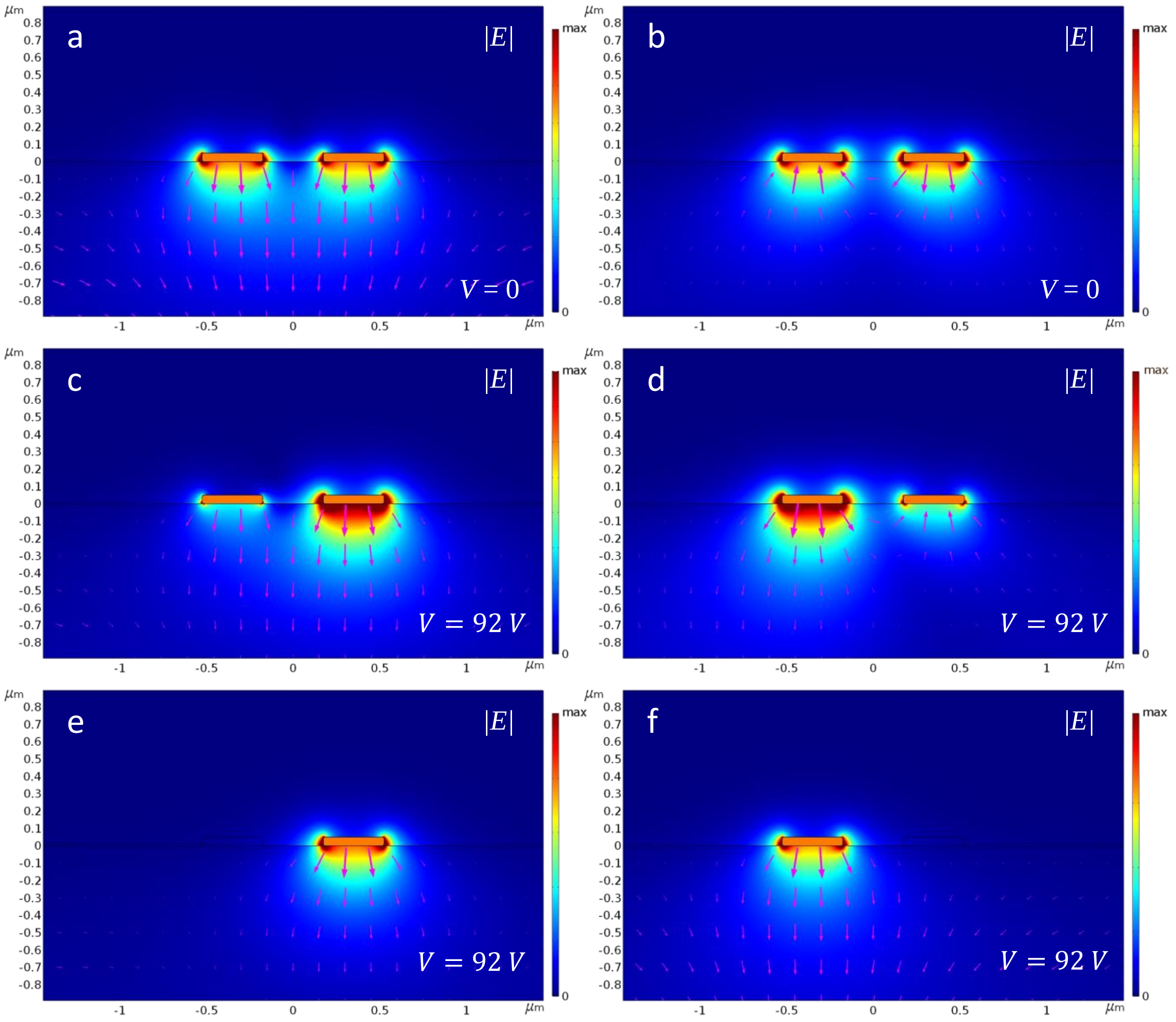

Supplementary Figure 3. (a, b) Optical $|E|$-field distributions for (a) even and (b) odd modes, when no voltage is applied. **(c-f)** Optical $|E|$-field distributions for (c) even, (d) odd, and (e,f) modes of individual waveguides upon applied full-switch voltage ($V = 92$ V). Gold waveguides are marked in orange. Magenta arrows represent transverse components of the optical E-field. The even mode localizes more in the region with increased refractive index, resulting in the increase of its effective mode index upon applied bias voltage. On the contrary, the odd mode has the opposite tendency.

In order to calculate the modification $\beta(V) - \beta(V = 0)$ of the propagation constants of each individual mode as a function of the applied voltage, we consider a single metal stripe in our model (Supplementary Figures 3e,f), and used the modified refractive index of LN substrate determined in the previous electrostatic step. Additionally, to prove the predictions of CMT, we run the mode analysis with both waveguides present and find modified even and odd modes (Supplementary Figures 3c,d). The strong asymmetry of even and odd mode distributions, predicted by CMT (with the asymmetry coefficient $\alpha = 1 \pm \sqrt{2}$ at full switch-off voltage), is clearly verified as shown in Supplementary Figures 3c,d.

The results of optical simulations are summarized in Supplementary Figure 4 below. As can be seen in Supplementary Figure 4b, using clamped values of LN permittivity reduces the modulation by ~20%, as compared to the modulation, calculated with unclamped values of LN permittivity. Overall, the CMT predicted effective mode indices are in good agreement with ones found in the numerical simulations.

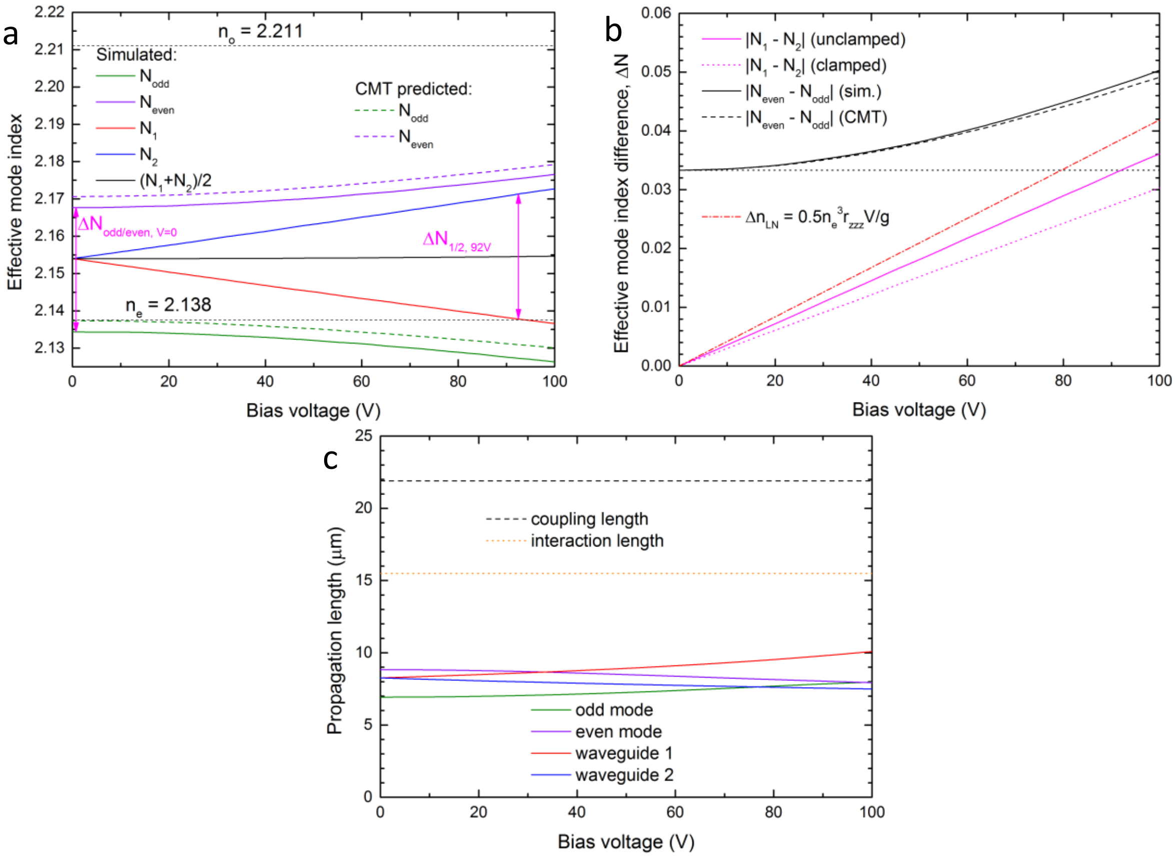

Supplementary Figure 4. a Effective mode index as a function of the applied bias voltage. Predictions from CMT are plotted with dashed lines. **b** Difference in the effective mode indices of individual waveguides ($|N_1$-$N_2|$, magenta lines), and of even and odd modes ($|N_{even}$ - $N_{odd}|$, black line) as a function of the applied bias voltage. Predictions from CMT are plotted with dashed lines. Full switch condition is achieved in our device when $|\Delta\beta_n| = 1$, i.e., when the electrically-induced difference in effective mode indices of individual waveguides, $|N_1$-$N_2|$ (magenta solid line), is equal to the difference in the effective mode indices of even and odd modes at no applied voltage, $|N_{even,V=0}$ - $N_{odd,V=0}|$ (black dotted line). This is satisfied at the bias voltage of ~92 V. Magenta dotted line shows calculations when using clamped values of LN permittivity; the rest is calculated with unclamped values of LN permittivity. Red dash-dotted line shows the expected average electrically-induced change in the refractive index of LN, assuming average electrostatic field as $E_z = V/g$, where $g = 350$ nm is the edge-to-edge separation of waveguides. **c** Propagation length as a function of the applied bias voltage for individual waveguide modes and even/odd supermodes. Black dashed line shows the

coupling length L_C; orange dotted line shows the optimal interaction length $L = L_C/\sqrt{2}$, required for a complete switch.

Finally, by comparing the effective mode index modulation $|N_1\text{-}N_2|$ and the expected average electrically-induced change in the refractive index of LN Δn_{LN} (assuming average electrostatic field as $E_z = V/g$, where $g = 350$ nm is the edge-to-edge separation of waveguides), one can calculate the interaction factor Γ, which quantifies the strength of the nonlinear electro-optic interaction of modulating field and optical mode:[6,7,8]

$$\Gamma \equiv \frac{|N_1 - N_1|}{\Delta n_{\mathrm{LN}}} = \frac{\lambda g}{V_\pi L n_{\mathrm{e}}{}^3 r_{zzz}}, \tag{2.1}$$

where L is the optimal interaction length, $L = L_C/\sqrt{2}$. We found that in our configuration it is $\Gamma \approx 0.86$, meaning quite a good overlap between electrostatic (RF) and optical fields.

Supplementary Note 3: Influence of free-space wavelength

In this section we study how the chosen free-space wavelength influences the modulator performance. For this the simulation domain was the same (350×50 nm^2 Au waveguides, separated by 350-nm gap), and the wavelength was varied. The Pockels coefficients of LN were kept constant. Since the modulation $\Delta\beta$ is linearly proportional to the applied voltage (as verified in Supplementary Figure 4b), here we did simulations only for the voltage of 0 and 50 V. At 0 V we calculated effective mode indices of even and odd mode in order to find the coupling length L_C and required modulation for the complete switch $\Delta\beta = 2[\beta_{\text{even}}(V=0) - \beta_{\text{odd}}(V=0)]$. For single-waveguide simulations (when the other waveguide is set to air) we did simulations at 0 and 50 V to find $\Delta\beta_{50\text{ V}}$, which we used to calculate the required full switch-off voltage

$$V_{\text{full switch}} = 2 \cdot [50\text{ V}] \frac{\beta_{\text{even}}(V=0) - \beta_{\text{odd}}(V=0)}{\Delta\beta_{50\text{ V}}} \tag{3.1}$$

We plot two figures of merit: 1) drive voltage-length product $VL = V_{\text{full switch}} L_C/\sqrt{2}$, and 2) normalized drive voltage-length product VL/L_{prop}, where the normalization is done over the propagation length (calculated for single waveguide at zero applied voltage).

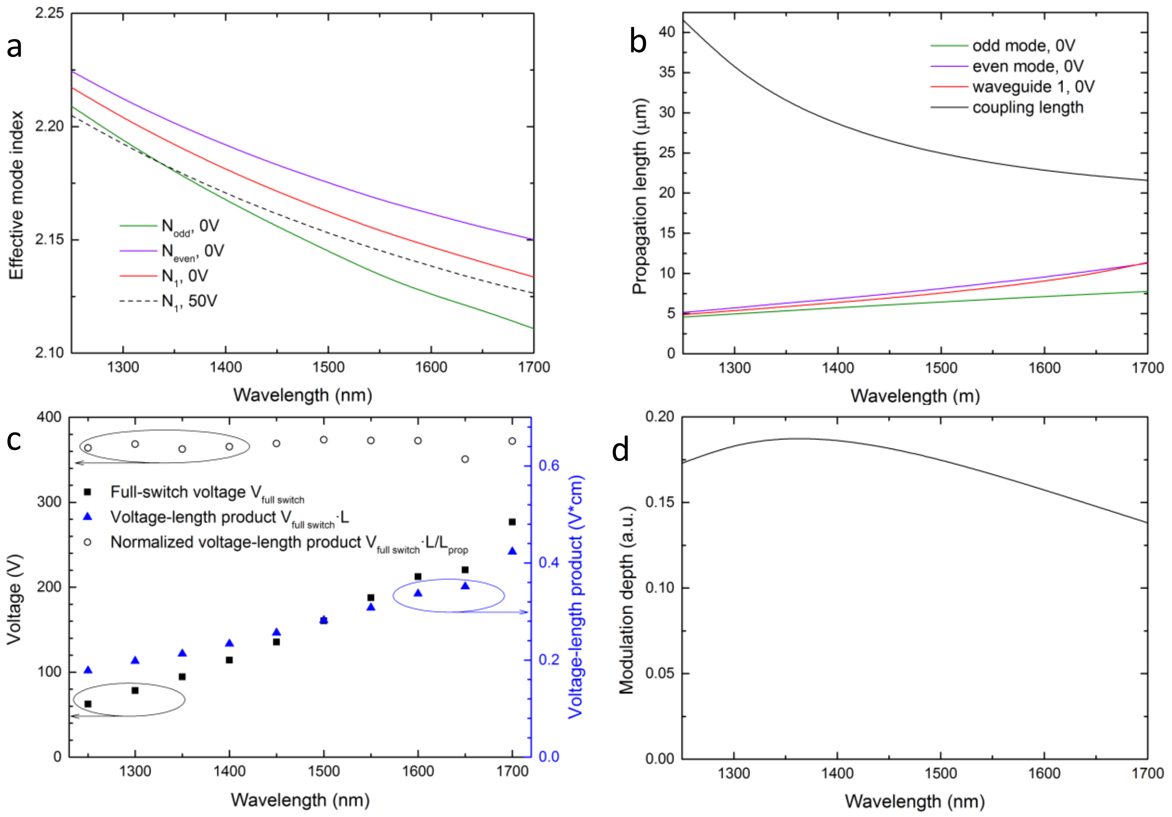

Supplementary Figure 5. a Wavelength dependence of the effective mode index of even (violet), odd (green), and mode of the individual waveguide (red) at zero applied voltage, compared with the effective mode index of individual waveguide mode at the applied voltage of 50 V (black dashed line). **b** Propagation length as a function of the wavelength for individual waveguide mode (red) and even/odd supermodes (violed and green,

respectively). Black line shows the coupling length L_C. **c** Estimated full-switch voltage $V_{\mathrm{full\,switch}}$ (black filled squares), voltage-length product $V_{\mathrm{full\,switch}}L$ (blue filled triangles), and voltage-length product, normalized to the propagation length, $V_{\mathrm{full\,switch}}L/L_{\mathrm{prop}}$ (black hollow circles). **d** Wavelength dependence of the modulation depth of the same device, optimized at λ = 1550 nm, at small driving voltages.

One can see in Supplementary Figure 5a that for a shorter wavelength, the modulation (change in N_1) is higher due to the higher refractive index of LN (since electro-optic modulation Δn is proportional to n^3), and due to the higher mode confinement (optical mode distribution like the one on Supplementary Figure 2e is more confined to the gold/LN interface, where the electro-optically induced change of the refractive index is highest). At the same time the difference between even and odd mode indices decreases with the decrease of the wavelength. Consequently, the coupling length increases at shorter wavelengths (and thus the full-switch device length) (Supplementary Figure 5b), but the required full-switch voltage becomes smaller (Supplementary Figure 5c). The voltage-length product is also smaller for shorter wavelengths, but when normalized to the propagation length, VL/L_{prop} becomes nearly constant for all investigated wavelengths (Supplementary Figure 5c), because the propagation length decreases substantially with a decrease of the wavelength (Supplementary Figure 5b). Considering the voltage-length-loss product, there is no significant benefit in changing the operation wavelength band.

Finally, we investigate the wavelength dependence of the modulation depth of our device, which was optimized for the free-space wavelength of λ = 1550 nm (reported also as Figure 4b in the main text). From Eq. 1.15 it follows that for small modulation ($|\Delta\beta_\mathrm{n}| \ll 1$) the modulation depth is

$$\mathrm{MD}(\lambda) = \frac{P_1(V)-P_1(V=0)}{P_1(V=0)} \approx \Delta\beta_\mathrm{n}(\lambda)\sin^2\left(\frac{\pi}{2}\frac{L}{L_\mathrm{C}(\lambda)}\right) \propto \Delta\beta(\lambda)L_\mathrm{C}(\lambda)\sin^2\left(\frac{\pi}{2}\frac{L}{L_\mathrm{C}(\lambda)}\right), \tag{3.2}$$

where $L = L_\mathrm{C}(\lambda = 1550\ \mathrm{nm})/\sqrt{2}$ is interaction length in our device. We plot the last expression in Eq. 3.2 in Supplementary Figure 5d, while the exact solution for a modulation voltage of 15 V is plotted in Figure 4b of the main text (in dB scale and normalized to the value at our operation wavelength of 1550 nm). The product of the first two terms in Eq. 3.2 [i.e., $\Delta\beta(\lambda)L_\mathrm{C}(\lambda)$] is inversely proportional to the voltage-length product, therefore it causes the increase of the modulation depth with the decrease of the voltage. However, the last sine term in the above equation decreases with the decrease of the wavelength (resulting in the increase of the coupling length), therefore there is an extremum (maximum) at the wavelength where the above factors cancel each other out.

Supplementary Note 4: Fabrication tolerance analysis of the investigated coupler switch

The influence of structural deviations induced by fabrication uncertainties on the switching performance in the proposed directional coupler is investigated. Therefore, a fabrication tolerance analysis at a fixed driving voltage of V_{bias} = 15 V is conducted by varying the two most critical design parameters, i.e., waveguide separation and waveguide width, affecting the switching performance. Though the variation of the modulation depth depends on the driving voltage, it is sufficient in telecommunication applications to operate the modulator at a modulation depth of ~ 25 %, which is achieved at the driving voltage of 15 V. Supplementary Figure 6a shows the influence on the modulation depth in terms of deviation of the waveguide separation distance. The gap dimensions between two coupling waveguides primarily affects the coupling length, and thus $L_{\text{n}} = L/L_{\text{c}}$ introduced in the description of the coupled mode formalism in Supplementary Note 1, which is found to have an optimum value of $L_{\text{n}} = \sqrt{2}$ for highest modulation depth at a fixed interaction length L. The fact that the maximum depth is observed at slightly smaller separation than $g = 350$ nm (the optimum design conditions at $L = 15.6$ nm) is due to a stronger electrostatic field at smaller separation distance, which results in a larger electro-optically induced phase mismatch in the two waveguide arms. Overall, the variation in the modulation depth is fairly small, varying less than 8% over a separation distances from 250 to 450 nm. By investigating deviations of the waveguide width w, while keeping the separation distance fixed at $g = 350$ nm, a steady increase of the modulation depth widths is observed for smaller waveguides (Supplementary Figure 6b). This behavior can be explained by the local enhancement of both, the optical and electrostatic field, for smaller waveguide widths, which results in an increased phase mismatch and thus higher modulation depth. However, smaller waveguide width results in higher losses of the whole device, therefore we chose w = 350 nm as a compromise between high losses and low modulation depth. Details on the design optimization can be found in Supplementary Note 5. The inset in Supplementary Figure 6b shows exemplarily the enhanced electrostatic field at smaller waveguide width (w = 250 nm), inducing a stronger modification of the refractive index, compared to a larger waveguide width (w = 450 nm). Within a waveguide width deviation of $\Delta w =$ 100 nm, the modulation depth varies less than 10 %. Generally, the symmetrically fed directional coupler configuration exhibits more relaxed fabrication constraints than Mach-Zehnder or resonator-based modulators[9].

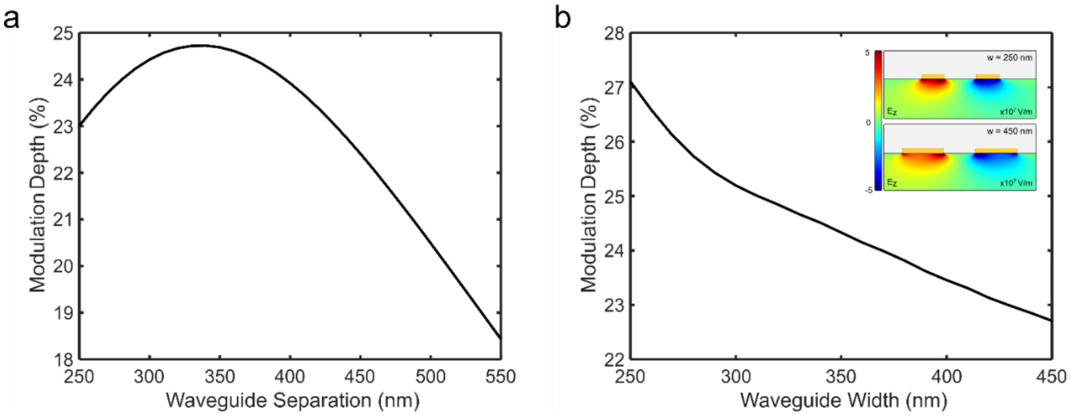

Supplementary Figure 6. Parametric geometric tolerance analysis of the directional coupler switch at V_{bias} = 15 V driving voltage. **a** Modulation depth versus waveguide separation with the fixed waveguide dimensions of 350 × 50 nm^2. **b** Modulation depth versus waveguide width with the fixed separation distance of 350 nm.

Supplementary Note 5: Design optimization for dual-channel phase modulators and directional coupler switches

In this section, a numerical optimization of the cross-section design is presented first in the general context of a dual-channel phase modulator. Since its practical implication is not restricted to the directional coupler switch (other device platforms like Mach-Zehnder interferometers or resonators are possible), we investigate the influence on the cross-section geometry on the half-wave voltage-length product for a phase shift of π between the two waveguides, the plasmonic losses and its interplay as voltage-length-loss product ($\mathrm{FOM} = V_\pi L\alpha$). For simplicity, the separation distance between the two wires is fixed at 350 nm as in our experimentally realized device. By reducing the waveguide dimension, the voltage-length product $V_\pi L$ can be reduced to below 0.2 V·cm (Supplementary Figure 7a). This is due to the tight optical confinement in smaller waveguides which enhances the light-matter interaction of the propagating mode (i.e. the mode is more confined close to the stripe where the refractive index change is largest). Contrarily, as the mode confinement becomes tighter, propagation losses are increasing for smaller waveguide dimensions (Supplementary Figure 7b). For waveguide-based device platforms in which the total optical losses should be kept reasonably small, an optimal trade-off between electro-optic efficiency (Supplementary Figure. 7a) and loss (Supplementary Figure 7b) must be found. Therefore, by multiplying the figures of merit, we defined the voltage-length-loss product $V_\pi L\alpha$ (Supplementary Figure 7c). Since the losses decrease faster than $V_\pi L$ increases with larger waveguide dimensions, we observe a steady decrease of the voltage-length-loss product for larger waveguide dimensions. Nevertheless, for each waveguide width one can find an optimal waveguide thickness in which best device performance regarding $V_\pi L\alpha$ is found, indicated as red line in Fig. S7c. The used waveguide geometry in our experiment (marked as black diamond in Supplementary Figure 7) is close to this optimal condition.

In our directional coupler modulator, another relevant parameter that dictates the device performance is the coupling length L_c (Supplementary Figure 7d). As described in Supplementary Note 1, the optimal interaction length is $L = L_c\sqrt{2}$. By multiplying the optimum interaction length with the propagation loss α, one can determine the insertion loss of the directional coupler (Supplementary Figure 7e). In our study, we target a small voltage-length product with an acceptable optical insertion loss which is kept below 6 dB, thus exhibiting comparable insertion losses as in commercially available electro-optic modulators[10]. By keeping the waveguide thickness at 50 nm and further decreasing the waveguide width, the voltage-length product can be reduced while the insertion loss (due to plasmonic propagation) is kept below 6 dB, since the optimum interaction length scales with the reduced coupling length. However, this only provides minor performance improvements and introduces potential performance fluctuations due to fabrication imperfections (i.e. stronger thickness dependence of $V_\pi L$ and α at smaller waveguide width). Furthermore, the maximum extinction ratio is limited in lossy (plasmonic) directional couplers, when $L_c \gtrsim L_{\mathrm{prop}}$, due to the difference in propagations

losses in even and odd mode. The maximum extinction ratio in the conventional passive directional coupler can be defined by[11]

$$\mathrm{ER_{max}} = \max(P_1/P_2) = \{\tanh(\mathrm{Im}[\Delta n]k_0 L_c)\}^{-2} \quad (5.1)$$

where the difference in the effective mode indices of the odd and even mode is $\Delta n = n_{odd} - n_{even}$, k_0 is the free-space wave vector and L_c is the coupling length. Our passive directional coupler device is expected to have a maximum extinction ratio of more than 20 dB (Supplementary Figure 7f), which is well below the electro-optically induced extinction ratio of 10 dB in the active device.

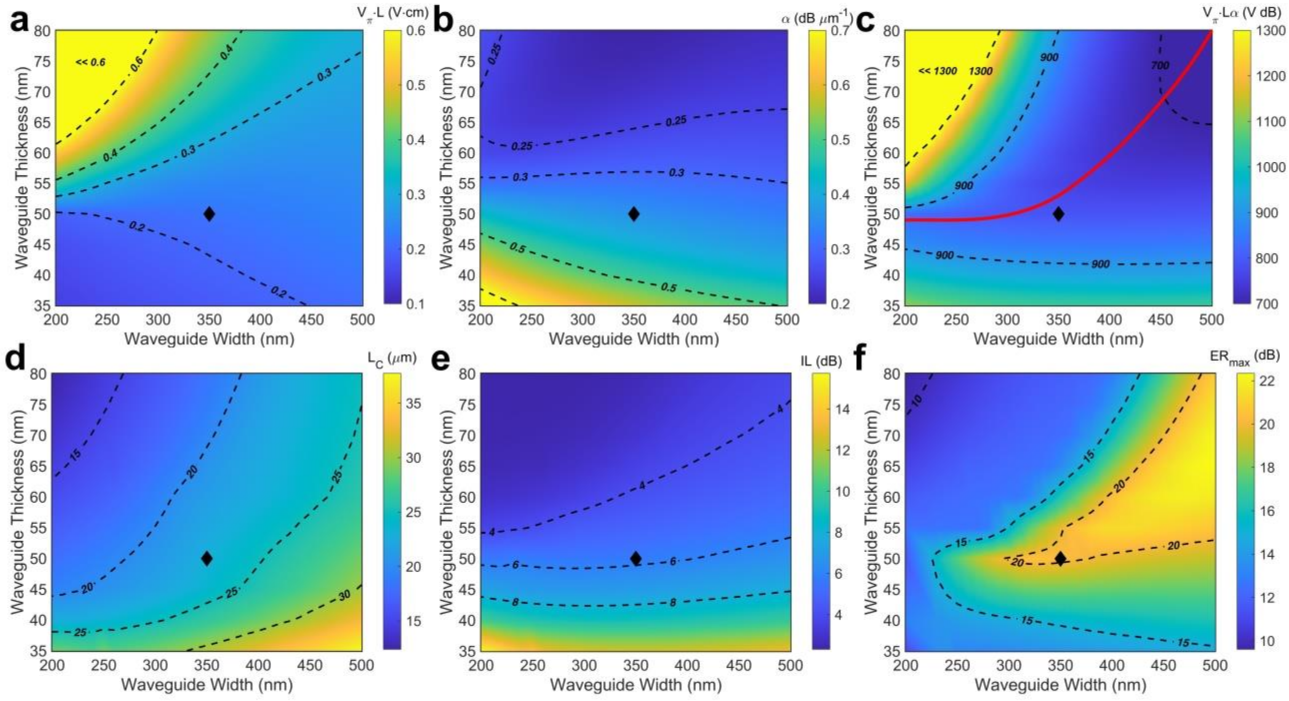

Supplementary Figure 7. (a-c) Design optimization for the plasmonic phase modulator. Figures of merit (**a** Half-wave voltage-length product $V_\pi L$, **b** propagation loss α, **c** voltage-length-loss product $V_\pi L\alpha$) are presented as a function of the waveguide dimensions with fixed waveguide spacing of 350 nm. The red line in **c** indicates the optimal width-thickness ratio where the smallest voltage-length-loss product can be found. **(d-f) Parametric study of the plasmonic directional coupler. d** Coupling length, **e** insertion loss (IL) and **f** maximum extinction ratio (ER) as a function waveguide width and thickness of a directional coupler with fixed separation distance of 350 nm. The black diamond shows the design parameters used in this work (350 × 50 nm^2).

Supplementary Note 6: Device capacitance and power consumption

By performing 3D-FEM simulations, the capacitance of the device with an interaction length of 15.5 um was found to be 3.6 fF, which dictates the capacitance-limited modulation bandwidth to be approximately 800 GHz with a 50 Ω source impedance. The calculated capacitance for different device lengths is shown in Supplementary Figure 8a. The energy consumption as a function of the modulation depth can be estimated according to[17]:

$$E = \frac{1}{4} C V_{\text{bias}}^2 , \tag{6.1}$$

where C is the device capacitance of the directional coupler and V_{bias} is the driving voltage. The calculated energy consumption as a function of the modulation depth translated from the modulation voltage is shown in Supplementary Figure 8b. Considering that most applications require relatively low modulation depth, operation with an energy consumption below 1 pJ bit^{-1} is feasible, which is smaller than many silicon-based photonic modulators.

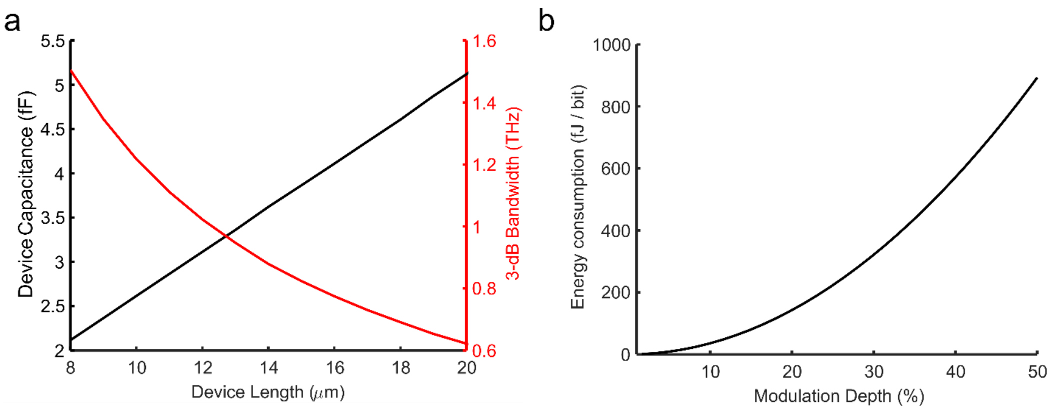

Supplementary Figure 8. Simulated electric-optical characteristics of the directional coupler modulator **a** Device capacitance and 3-dB bandwidth of the directional coupler modulator with a 50 Ω source impedance **b** Energy consumption as a function of the modulation depth.

Supplementary Note 7: Experimental set-up for measuring the modulation characteristics

A far-field optical setup (Supplementary Figure 9) is used for characterizing the electro-optic modulator. A tunable IR telecom laser is providing a collimated laser beam with a selectable wavelength. After the beam is periodically interrupted with the frequency f_c by an optical chopper (optional for the RF measurement), its polarization is defined by a polarizer (Pol) and a half-wave plate (HWP). The beam (p-polarization) passes through a polarizing beam splitter and IR-objective (× 100 magnification, NA=0.95). The focused diffraction-limited beam excites the plasmonic mode by precisely positioning the device under test (DUT). The incident beam is symmetrically illuminating the input grating coupling, in a way that the power at the output grating ports collected by the objective is equally distributed. The polarization of the emitted signal from the terminating coupling gratings is rotated by the waveguide bend to be orthogonal to the polarization of the incident beam and thus allowing suppression of back-reflections from the incident beam by the polarizing beam splitter. The signal is spatially filtered at the image plane to extract power only from one of the output ports. Using a fiber collimator, the signal is fiber-coupled and detected by an InGaAs high-speed photodetector. For electro-optically modulation, the driving voltage with frequency f_m is applied to the plasmonic structure, by mechanical clamping of electric probes on the connecting electrode pads of the chip. The wavelength- and voltage-dependent modulation depth is measured with an oscilloscope. For the high-speed measurement, the modulated signal is measured with a spectrum analyzer (SA), where the electro-optically modulated optical signal is observed at the intentionally shifted frequency caused by modulating the signal with the optical chopper. In this way the chopper modulation generates sidebands at $f_m \pm f_c$, which are contrary to the carrier signal free of electrical noise induced by electrical crosstalk between signal generator and spectrum analyzer (see Supplementary Note 8).

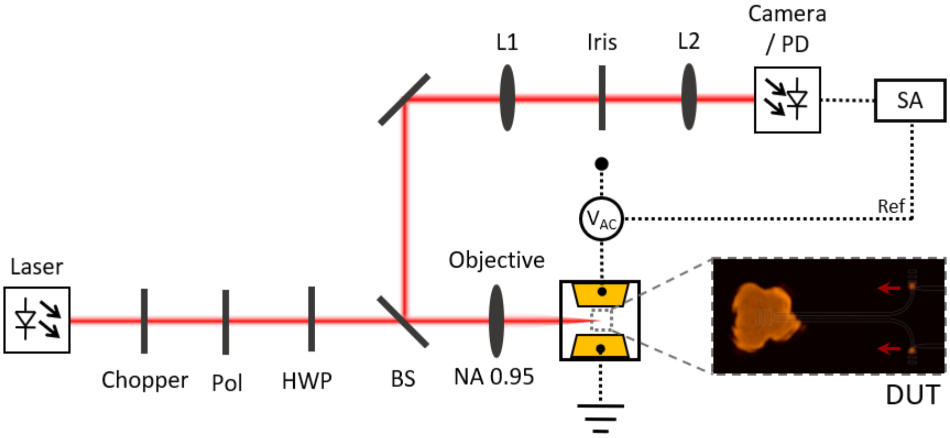

Supplementary Figure 9. Experimental set-up for characterizing the plasmonic modulator. The optical path is illustrated as red lines and the electrical wiring as dashed black lines.

Supplementary Note 8: RF characterization

The limited sensitivity of the photodetector (Thorlabs PDA8GS) and the fact that we did not include an optical amplifier in the detection path resulted in an RF transfer function of the overall system of as low as -140 dB. We did not have access to a vector network analyzer of the required bandwidth and dynamic range. Instead, we measured the RF response by exciting the modulator with a microwave synthesizer (HP8672S, excitation level approx. +10 dBm) and detecting the photocurrent with a separate RF spectrum analyzer (Rohde&Schwarz FSM, noise floor approx. -150 dBm at 6 Hz resolution bandwidth).

The plasmonic modulator was placed at the end of a coplanar waveguide composed of a 150 nm thick gold wire on the $LiNbO_3$ substrate with a characteristic impedance of 50 Ω +- 20%, a length of approx. 5 mm and an Ohmic resistance of 20-30 Ω. This waveguide was terminated by two thin gold wires (50 nm thick, designed to be 100 Ω each) fabricated together with the modulator. The termination wire resistance was subject to considerable variation from sample to sample leading to tolerable ripple in the RF-response.

On the opposite end, the coplanar waveguide was contacted by a low-profile RF-probe that was made in-house specifically to fit in the working distance of the microscope objective. It involved three contact tips, which formed a 2 mm short poorly matched coplanar waveguide (approx. 200 Ω characteristic impedance). Finally, the optical signal was detected with a commercial photoreceiver module connected directly to the spectrum analyzer. The detector is specified with a bandwidth of 9.5 GHz and according to the vendor has a first-order low-pass response.

The low RF signal at the receiver led to significant problems with direct crosstalk due to leakage from RF cables and through power cords. Because of the strong background signal at the microwave stimulus frequency and the sidebands at the harmonics of the power grid frequency, we modulated the optical incident power with a chopper at 75 Hz introducing RF-sidebands that were an unambiguous signature of the optical modulator.

We calibrated the frequency dependence of the stimulus power level caused by variations in the synthesizer and the cablework by replacing the RF contacts with a bolometric power detector (HP8481B). Furthermore, we subtracted the typical response of the photodetector (first-order low-pass pole at 9.5 GHz) from the total signal. Since we did not have access to a vector network analyzer or a reflectometer with the necessary bandwidth, we could not calibrate out the RF response of the contact pads and the on-chip waveguide. As an alternative, we simulated it using designed (characteristic impedance of the waveguide and the contact tips) and measured parameters (Ohmic resistance of the waveguide and the termination resistor). The resulting RF-response is shown in Supplementary Figure 10 alongside an equivalent schematic. We find that this RF model is in excellent agreement with the measured data (both shown in Fig. 4c of the main text). We stress that the model was not fitted to the measured data but was based on unrelated measurements. Therefore, we are confident that the apparent drop in modulated signal at higher frequencies is due to imperfections in the RF

contacts and feedline. We do not observe any bandwidth limitation from the plasmonic modulator itself as expected from our earlier estimates based on the device's capacitance.

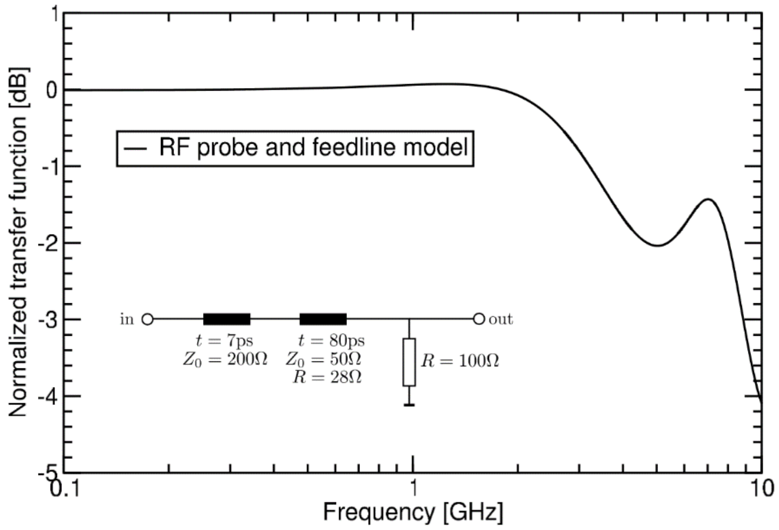

Supplementary Figure 10. The resulting RF response of the designed (characteristic impedance of the waveguide and the contact tips) and measured parameters (Ohmic resistance of the waveguide and the termination resistor) in our experiment, alongside an equivalent schematic (inset).

Supplementary Note 9: Near-field investigation of the directional coupler

We study the near-field evolution of directional coupling using an AFM-based phase-resolved scattering-type scanning near-field optical microscope (SNOM from NeaSpec) operated in a transmission configuration[12-15]. A commercial Si probe, coated by PtIr alloy (Arrow NcPt, NanoWorld), is used in a non-contact tapping mode (tapping frequency ~250 kHz, tapping amplitude ~50 nm). Scattering from this probe, measured by an amplified InGaAs detector, is demodulated at third harmonic of the tapping frequency in order to get rid of a strong background (bulk scattering from the tip, the cantilever, and the sample itself, which is not modulated by the tip-sample distance). The setup additionally employs a Mach-Zehnder interferometer with modulated phase of the reference arm (by means of mirror oscillations at a frequency ~300 Hz), which allows using a pseuhederodyne demodulation[16] to resolve both the amplitude and the phase. As a source, we used a telecom laser (New Focus Venturi TLB-6600) at CW output (λ = 1550 nm, ~8 mW), which was focused onto the sample (spot FWHM by intensity ~3.5 μm, corresponding to the NA ~0.16) normally from below by a custom-made right-angle parabolic mirror.

In order to investigate directional coupling and measure the coupling length accurately, we fabricated directional couplers with input into one of the channels (Supplementary Figure 11a), which equally excites even and odd modes. The fabricated directional coupler on *z*-cut LN incorporates a tapered grating which transforms free-space light from a loosely focused beam into a single nanowire waveguiding mode. After propagating 3 μm through a single waveguide, an identical adjacent waveguide is introduced, enabling evanescent coupling between those two waveguides. The length of the directional coupler is chosen large enough to avoid back reflections from its termination. Shortly after scanning the fabricated devices we encountered a problem of sample charging, which prohibits the tip to come close to the sample surface, making it impossible to record proper topography and near-field signal. We believe it is because of a piezoelectric effect of LN, caused by the oscillating tip, therefore we deposited 10 nm of SiO_2 to suppress this effect. By numerical simulations we have verified that this additional layer does not significantly influence modal properties of the system (though additional losses might appear due to the scattering on the roughness, caused by this layer). The dashed white box in Supplementary Figure 11a indicates the scanned area. An AFM topography image of this area is shown in Supplementary Figure 11b, along with the recorded near-field maps of the amplitude and the phase (Supplementary Figures 11c-d). Noticeably when approaching the 3-dB coupling point, a $\pi/2$ relative phase shift between the throughput light and the coupled light is observed as expected for evanescent coupling between two closely spaced identical waveguides. The near-field data of the coupling section is first analyzed in the Fourier domain, and then fitted as a sum of propagating modes by a least square method[12-16]. The mode effective indices and propagation lengths of the odd and even modes are measured for three different coupler configurations and compared with numerical simulations (Supplementary Table 1). A movie of the evolution of the real-valued field is provided in Supplementary Movie 1.

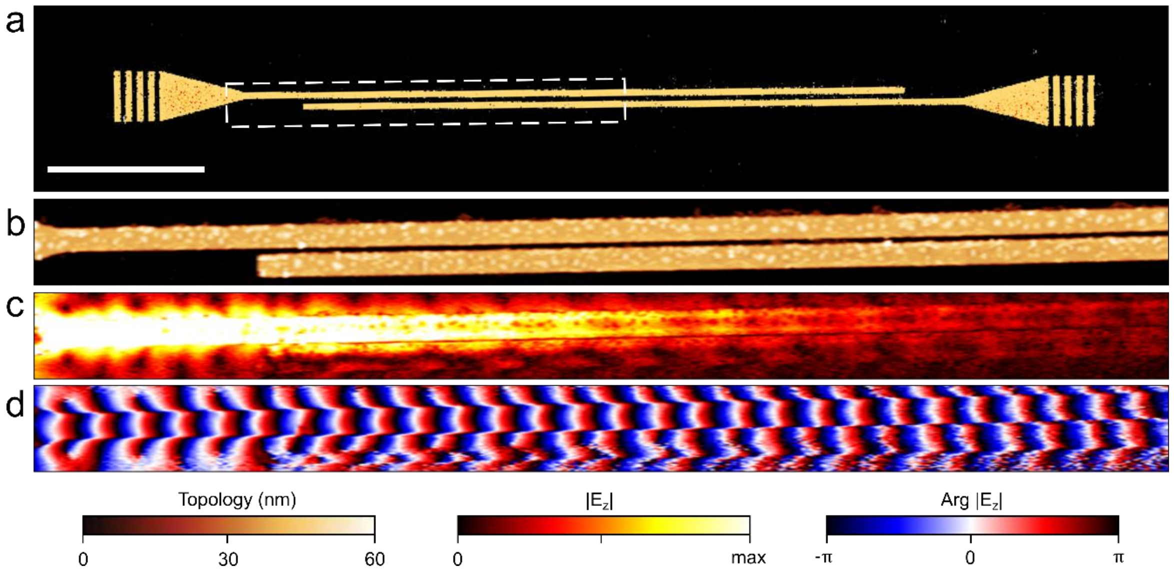

Supplementary Figure 11. Near-field investigation of the plasmonic directional coupler using phase-resolved scattering-type near-field optical microscopy (s-SNOM). **a** False-color scanning electron microscope (SEM) image of the investigated directional coupler with tapered grating coupler. A laser beam with the wavelength $\lambda_0 = 1550$ nm is positioned at the left input grating in order to couple optical power into one plasmonic waveguide arm. The dashed box indicates the area that was investigated using s-SNOM. The scale bar represents 10 µm. **b** Atomic Force Microscopy (AFM) topography of the investigated area, **c** near-field amplitude $|E_z|$, and **d** near-field phase $\text{Arg}[E_z]$, measured with s-SNOM.

**Waveguide dimensions**		**420×50 nm²**	**420×50 nm²**	**390×50 nm²**
**Separation**		**100 nm**	**200 nm**	**190 nm**
**Experiment**	n_{odd}	2.22±0.01	2.21±0.01	2.20±0.01
	n_{even}	2.12±0.01	2.15±0.01	2.15±0.01
	$L_{P,odd}$	4±2 μm	7±4 μm	8±4 μm
	$L_{P,even}$	1.2±0.5 μm	3±2 μm	3±2 μm
	L_C	8±2 μm	13±4 μm	14±6 μm
**Simulations**	n_{odd}	2.171	2.169	2.167
	n_{even}	2.119	2.122	2.119
	$L_{P,odd}$	14.2 μm	13.7 μm	13.1 μm
	$L_{P,even}$	8.6 μm	9.0 μm	9.7 μm
	L_C	14.9 μm	16.5 μm	16.1 μm

Supplementary Table 1. Experimentally obtained mode effective indices and propagation length of the odd and even mode, compared with numerical simulations.

Supplementary Note 10: Comparison with state-of-the-art electro-optic modulators

**Material Platform**	**r_{33} / pm·V^{-1}**	**Thermal stability**	**$V_\pi \cdot L$ / V · cm**	**Insertion loss / dB**	**Reference**
LN	30	+	9.4	1	[18]
LN	30	+	6.4	0.3	[19]
PZT	61	+/-	3.2	0.05	[20]
LN	30	+	2.2	0.4	[21]
LN	30	+	1.8	1.5	[22]
BTO	107	+/-	0.66	N/A	[23]
BTO	340	+/-	0.45	2.1	[24]
**LN (Plasmonic)**	**30**	**+**	**0.21**	**5.5**	**Our work**
EOP M3	91	-	0.11	2.5	[25]
EOP YLD-124 (Plasmonic)	100	-	0.012	6	[26]
EOP DLD-164 (Plasmonic)	180	-	0.006	2.5	[27]

Supplementary Table 2. Comparison of state-of-the-art electro-optic Pockels modulators sorted by the half-wavevoltage-length product. The material platform used in the individual modulator devices are lithium niobite (LN), lead zirconate titanate (PZT), barium titanate (BTO) and electro-optic polymers (EOP). The insertion loss is defined by the waveguide propagation loss in the phase-shifter section. The thermal stability is classified by the threshold temperature T_t of structural phase transition in the material to be low (-) at T_t < 100 °C, medium (+/-) at 100 °C < T_t < 300 °C or high (+) at T_t > 300 °C.

References

[1] Alferness, R. Guided-wave devices for optical communication. *IEEE J. Quantum Elect.* **17**, 946–959 (1981).

[2] Kogelnik, H. and Schmidt R. Switched directional couplers with alternating ΔB. *IEEE J. Quantum Elect.* **12**, 396–401 (1976).

[3] Jazbinšek, M. and Zgonik, M. Material tensor parameters of LiNbO3 relevant for electro- and elasto-optics. *Appl. Phys. B* **74**, 407–414 (2002).

[4] Zelmon, D. E., Small, D. L. and Jundt, D. Infrared corrected Sellmeier coefficients for congruently grown lithium niobate and 5 mol % magnesium oxide–doped lithium niobate. *J. Opt. Soc. Am. B* **14**, 3319-3322 (1997).

[5] Rakić, A. D., Djurišic, A. B., Elazar, J. M. and Majewski. M. L. Optical properties of metallic films for vertical-cavity optoelectronic devices, *Appl. Opt.* **37**, 5271-5283 (1998).

[6] Brosi J-M, Koos C, Andreani LC, Waldow M, Leuthold J, Freude W. High-speed low-voltage electro-optic modulator with a polymer-infiltrated silicon photonic crystal waveguide. *Opt. Express* **16**, 4177-4191 (2008).

[7] Witzens J, Baehr-Jones T, Hochberg M. Design of transmission line driven slot waveguide Mach-Zehnder interferometers and application to analog optical links. *Opt. Express* **18**, 16902-16928 (2010).

[8] Alexander, K. et al. Nanophotonic Pockels modulators on a silicon nitride platform. *Nat. Commun.* **9**, 3444 (2018)

[9] Tavlykaev, R. F. and Ramaswamy, R. V. Highly linear Y-fed directional coupler modulator with low intermodulation distortion. *J. Lightwave Technol.* **17**, 282–291 (1999).

[10] *Lithium Niobate Electro-Optic Modulators*. https://www.thorlabs.com (2019)

[11] Zenin, V. A., Volkov, V. S., Han, Z., Bozhevolnyi, S. I., Devaux, E. and Ebbesen, T. W. Directional coupling in channel plasmon-polariton waveguides. *Opt. Express* **20**, 6124-6134 (2012).

[12] Deshpande, R., Zenin, V. A., Ding F., Mortensen, N. A. and Bozhevolnyi, S. I. Direct Characterization of Near-Field Coupling in Gap Plasmon-Based Metasurfaces. *Nano Lett.* **18**, 6265-6270 (2018).

[13] DeVault, C. T., Zenin, V. A., Pors, A., Chaudhuri, K., Kim, J., Boltasseva, A., Shalaev, V. M. and Bozhevolnyi, S. I. Suppression of near-field coupling in plasmonic antennas on epsilon-near-zero substrates. *Optica* **5**, 1557-1563 (2018).

[14] Zenin, V. A., Choudhury, S., Saha, S., Shalaev, V. M., Boltasseva, A. and Bozhevolnyi, S. I. Hybrid plasmonic waveguides formed by metal coating of dielectric ridges. *Opt. Express* **25**, 12295-12302 (2017).

[15] Zenin, V. A., Malureanu, R., Radko, I. P., Lavrinenko, A. V. and Bozhevolnyi, S. I. Near-field characterization of bound plasmonic modes in metal strip waveguides. *Opt. Express* **24**, 4582-4590 (2016).

[16] Ocelic, N., Huber, A. and Hillenbrand, R. Pseudoheterodyne detection for background-free near-field spectroscopy. *Appl. Phys. Lett.* **89**, 101124 (2006).

[17] Miller, D. A. Energy consumption in optical modulators for interconnects. *Opt. Express* **20**, A293-A308, (2012).

[18] Mercante, A. J., Yao, P., Shi, S., Schneider, G., Murakowski, J., and Prather, D. W. 110 GHz CMOS compatible thin film LiNbO3 modulator on silicon. *Opt. Express* **24**, 15590–15595 (2016).

[19] Weigel, P. O. *et al.* Bonded thin film lithium niobate modulator on a silicon photonics platform exceeding 100 GHz 3-dB electrical modulation bandwidth. *Opt. Express* **26**, 23728-23729 (2018).

[20] Alexander, K. *et al.* Nanophotonic Pockels modulators on a silicon nitride platform. *Nat. Commun.* **9**, 3444 (2018).

[21] Wang, C. *et al.* Integrated lithium niobate electro-optic modulators operating at CMOS-compatible voltages. *Nature* **562**, 101–104 (2018).

[22] Wang, C., Zhang, M., Stern, B., Lipson, M., and Lončar, M. Nanophotonic lithium niobate electro-optic modulators. *Opt. Express* **26**, 1547-1555 (2018).

[23] Girouard, P., Chen, P., Jeong, Y., Liu, Z., Ho, S.-T., and Wessels, B. W. $\chi^{(2)}$ Modulator With 40-GHz Modulation Utilizing BaTiO3 Photonic Crystal Waveguides. *IEEE J. Quantum Elect.* **53**, 1–10 (2017).

[24] Abel, S. *et al.* Large Pockels effect in micro- and nanostructured barium titanate integrated on silicon. *Nat. Materials* **18**, 42–47 (2019).

[25] Alloatti, L. *et al.* 100 GHz silicon–organic hybrid modulator. *Light Sci. Appl.* **3**, e173 (2014).

[26] Ayata, M. *et al.* High-speed plasmonic modulator in a single metal layer. *Science* **358**, 630–632 (2017).

[27] Haffner, C. *et al.* All-plasmonic Mach–Zehnder modulator enabling optical high-speed communication at the microscale. *Nat. Photonics* **9**, 525–528 (2015).
