# Supplementary material for: Plasmonic monolithic lithium niobate directional coupler switches
Source: Nat Commun. 2020 Feb 6;11:748. doi: 10.1038/s41467-020-14539-y (PMC7005156; doi:10.1038/s41467-020-14539-y)
Supplement: Supplementary file 4 — Description of Additional Supplementary Files [file 41467_2020_14539_MOESM4_ESM.pdf]

Title: Supplementary movie 1: Temporal evolution of the propagating near-field in the directional coupler

Description: Near-field investigation of the plasmonic directional coupler using phase-resolved scattering-type near-field optical microscopy (s-SNOM). **a** False-color scanning electron microscope (SEM) image of the investigated directional coupler with tapered grating coupler. A laser beam with the wavelength $\lambda_0 = 1550$ nm is positioned at the left input grating couple optical power into one plasmonic waveguide arm. The dashed box indicates the area that was investigated using s-SNOM. **b** Measured Atomic Force Microscopy (AFM) topography of the investigated area, **c** near-field amplitude $|E_z|$, **d** near-field phase Arg$[E_z]$, and **e** evolution of the real value of E_z, i.e., Re$[E_z \exp(j\omega t)]$.

Title: Supplementary movie 2: Visualization of dynamic optical switching

Description: Optical power exchange at slow switching speeds (0.6 Hz) observed by an infrared camera **a** Colorized scanning electron microscope (SEM) image of the investigated plasmonic directional coupler switch. **b** The scattering signals at the output ports P1 and P2 are modulated by an oscillating driving voltage, revealing optical power exchange between the coupler waveguide arms. **c** Simulated electro-optical transfer function, illustrating the power exchange as a function of the applied bias voltage, synchronized with the experimentally observed power switching in **b**.
